# Rheology and Shape Stability Control of 3D-Printed White Calcium Sulfoaluminate Cement Composites Containing Oyster Shell and Cuttlebone Powder

**DOI:** 10.3390/ma19112410

**Published:** 2026-06-05

**Authors:** Xingyu Qu, Qinyuan Wang, Jiafeng Kong, Junyu Wang, Jie Wang, Xingang Xu, Yan Zheng, Heyang Wu, Mingxu Chen

**Affiliations:** 1 College of Civil Engineering & Architecture, Qingdao Agricultural University, Qingdao 266109, China; xyqu2002@163.com (X.Q.); 15064188200@163.com (J.W.); 18562577899@163.com (J.W.); chenmx@qau.edu.cn (M.C.); 2 Qingdao Product Quality Testing Research Institute, Qingdao 266061, China; mollywangqy@foxmail.com; 3 Centre for Future Materials, University of Southern Queensland, Toowoomba, QLD 4350, Australia; jiafeng.kong@unisq.edu.au; 4 State Key Laboratory of Silicate Materials for Architectures, Wuhan University of Technology, Wuhan 430070, China; xuxingang@whut.edu.cn

**Keywords:** 3D printing, white calcium sulfoaluminate cement, oyster shell powder, cuttlebone powder, rheological properties

## Abstract

To optimize the shape stability of 3D-printed white calcium sulfoaluminate (WCSA) cement composites, oyster shell powder (OSP) and cuttlebone powder (CBP) were introduced as white admixtures to regulate rheological properties and printability. The setting behavior, rheological properties, and shape stability of the WCSA cement composites were evaluated by Vicat setting-time tests, rotational rheological measurements, three-stage thixotropic recovery tests, and structural deformation measurements, together with mechanical strength tests, XRD, and SEM analyses. The results showed that the incorporation of OSP and CBP shortened the setting time of WCSA cement composites. The initial and final setting times decreased from 41 min and 67 min to 17 min and 30 min in the WCSA cement composites with OSP, and from 42 min and 66 min to 20 min and 33 min in the WCSA cement composites with CBP, which improved printing operability. As the OSP and CBP content increases from 0% to 24%, the dynamic yield stress of WCSA cement composites increased from 48.83 Pa to 530.59 Pa and 60.30 Pa to 1085.80 Pa, respectively. The thixotropic recovery degree of WCSA cement composites increased from 57.89% to 86.46%, and 56.60% to 92.14%, respectively. As the OSP and CBP contents increase from 0% to 24%, the structural deformation decreased from 12.39% to 6.91% and 13.29% to 5.12% respectively, which improved buildability of the printed structures. In addition, although OSP and CBP reduced the mechanical strength of WCSA cement composites compared with the control group, the flexural and compressive strengths gradually recovered as the contents increased from 6% to 24% due to the enhanced filling effect and improved particle packing. This study provides a reference for the application of marine calcareous solid wastes in sustainable 3D-printed cementitious materials.

## 1. Introduction

Extrusion-based 3D-printed cementitious materials require fresh mixtures to exhibit sufficient flowability, continuity, and extrudability during pumping and nozzle extrusion, while rapidly rebuilding the internal structure and developing adequate early-age bearing capacity after deposition [1,2,3]. These properties are essential for stable layer-by-layer stacking and the forming quality of printed structures [4,5]. Therefore, printable cementitious materials should maintain suitable flowability under shear and achieve rapid structural build-up at rest [6]. Yield stress, thixotropy, viscoelasticity, and early hydration behavior are key parameters controlling printability [7]. A low yield stress may lead to structural deformation or even collapse of printed filaments, whereas excessive structural build-up may shorten the printable window, resulting in discontinuous extrusion, nozzle blockage, and poor interlayer bonding [8]. Therefore, controlling the rheological parameters and structural rebuilding capacity of fresh mixtures is a critical issue for improving the shape stability and printing adaptability of 3D-printed cementitious materials.

White calcium sulfoaluminate (WCSA) cement is characterized by rapid setting, high early strength, and high whiteness, showing potential for rapid-forming and functional 3D-printed materials [9]. During early hydration, WCSA can rapidly generate ettringite (AFt)-dominated hydration products, which promotes the formation of a load-bearing skeleton within the paste and improves the structural build-up and interlayer stacking stability after deposition [10,11]. However, due to its high hydration activity, the rheological parameters of WCSA change rapidly with time, which may narrow the printable window, reduce extrusion continuity, and weaken interlayer bonding [12]. Xu et al. systematically investigated the rheological behavior, printed structures, and efflorescence control of 3D-printed white Portland cement-based materials, indicating that admixtures can improve the printability of white cementitious materials by regulating rheological parameters and early structural formation [13]. Jin et al. further investigated the effect of accelerators on 3D-printed white cementitious materials and reported that the coupling between early hydration and rheological behavior directly determines the initial printability and structural build-up process [14].

Oyster shell powder (OSP) and cuttlebone powder (CBP) are typical waste marine biogenic calcareous powders, with calcium carbonate as their main mineral component [15]. However, their particle morphology and pore structure are significantly different from those of industrial limestone powder. OSP has a rough surface and irregular particle morphology, which may affect particle packing and early hydration through the filler effect, mechanical interlocking, and heterogeneous nucleation [16,17,18]. In contrast, CBP, characterized by its hierarchical porous structure and open channels, modifies the distribution of free water in fresh paste via water absorption and release, thereby yielding local lubrication under shear and thickening or internal-curing effects at rest [19,20]. Seifu et al. investigated the role of OSP in PC-CSA–slag and PC-CSA–metakaolin ternary systems and found that OSP promoted the formation of monocarboaluminate and hemicarboaluminate, and improved the stability of ettringite [21]. Čadež et al. reported that cuttlebone consists of hierarchically ordered aragonitic calcium carbonate structures, with adult samples containing approximately 100 parallel chambers, each with a height of about 600 μm [22]. Related studies also indicated that its inorganic phase is mainly aragonitic CaCO_3_ with a certain amount of organic matter. These characteristics suggest that OSP and CBP may not only act as fillers but also serve as rheology-controlling components that affect the yield stress, thixotropy, and structural build-up of WCSA pastes.

At present, studies on 3D-printed CSA cement-based materials have been conducted from different perspectives. Chen and Liu et al. investigated the durability, rheological properties, and structural build-up of 3D-printed cementitious materials [23,24]. Tao and Hu et al. studied the effects of CSA cement on binder-system design, printability, and early strength development [25,26]. Their mechanisms are generally attributed to thickening, filling, hydration regulation, or reinforcement effects. However, for WCSA cement, the effects of marine biogenic calcareous powders on rheological evolution, setting and hardening behavior, structural rebuilding, and mechanical properties remain unclear. In particular, during the non-equilibrium process of extrusion, deposition, resting and stacking, how the particle morphology, pore structure, and mineral composition of OSP and CBP affect the static yield stress, thixotropy, viscoelastic behavior, and hardened microstructure of WCSA cement-based materials has not been fully clarified.

In this study, OSP and CBP were selected as marine biogenic calcareous powders to investigate their effects on the rheological behavior of 3D-printed WCSA cement pastes. Mechanical tests, X-ray diffraction, and scanning electron microscopy were conducted to analyze the regulatory mechanisms of different powders on hydration products, microstructure, and strength development. This study aims to establish the relationship among powder morphology, rheological parameter evolution, early structural build-up, and mechanical properties, thereby providing a reference for rheology control and high-value utilization of marine solid wastes in sustainable 3D-printed WCSA based materials.

## 2. Materials and Methods

### 2.1. Raw Materials

**Figure 1:**
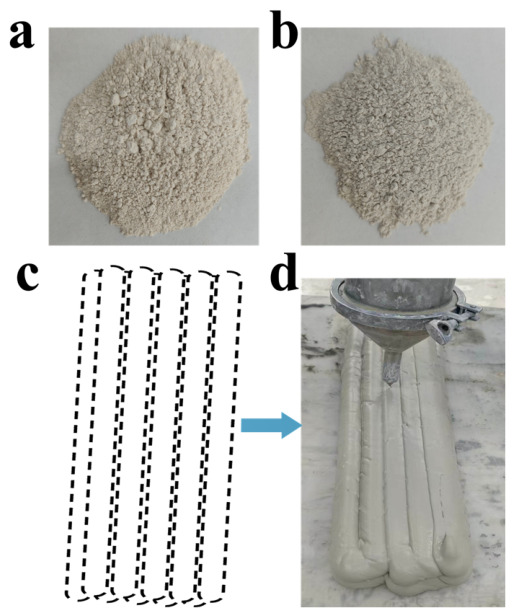
Photographs and schematic diagram of raw materials and printed samples: (**a**) OSP; (**b**) CBP; (**c**) schematic diagram of printed layers; (**d**) fresh mixture.

The studied 3D-printed white calcium sulfoaluminate cement composites comprised six components: white calcium sulfoaluminate cement (WCSA, Zhonglian Cement Co., Ltd., Beijing, China), tartaric acid (TA, analytical reagent, Macklin Biochemical Technology Co., Ltd., Shanghai, China), water-reducing agent (WRA, polycarboxylate, water-reducing rate of 32%, Shandong Provincial Academy of Building Research, Jinan, China), hydroxypropyl methylcellulose ether (HPMC, Shandong Heda Co., Ltd., Zibo, China), bentonite (Macklin Biochemical Technology Co., Ltd., Shanghai, China), and 100-mesh quartz sand (Gongyi Hengxin Filter Material Factory, Zhengzhou, China). Oyster shell powder (OSP) and cuttlebone powder (CBP) were introduced to regulate the rheological properties, structural build-up, and mechanical performance of the 3D-printed WCSA cement composites. OSP and CBP were obtained by washing, drying, crushing, grinding, and sieving oyster shells and cuttlebones through a 200-mesh sieve. The main raw materials, printed layers, and fresh mixture extrusion process are shown in Figure 1.

**Figure 2:**
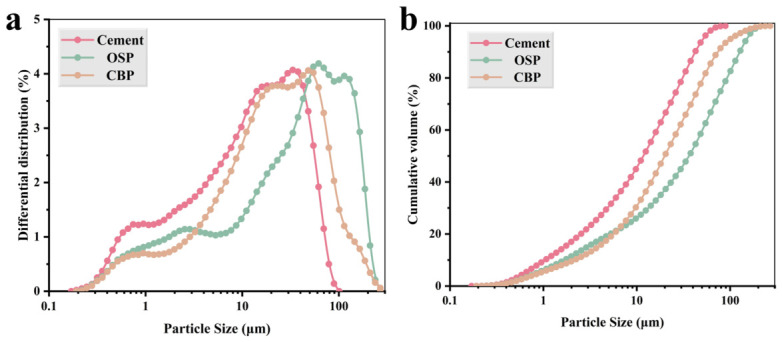
Particle size distribution of WCSA, OSP and CBP: (**a**) differential distribution; (**b**) cumulative distribution.

**Figure 3:**
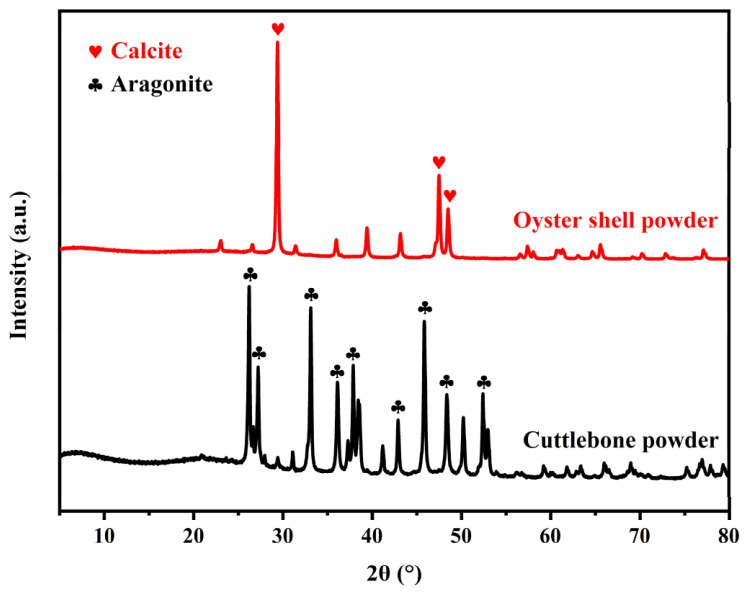
XRD patterns images of OSP and CBP.

**Figure 4:**
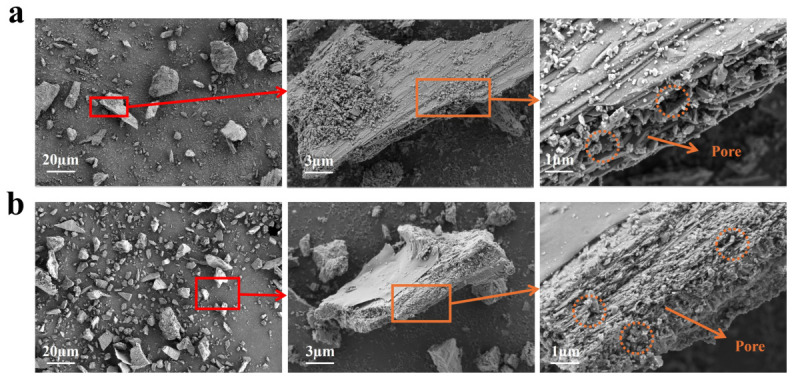
SEM images of marine calcareous powders at different magnifications: (**a**) OSP and (**b**) CBP.

Figure 2 shows the particle size distributions of WCSA cement, OSP, and CBP. Compared with WCSA cement, OSP and CBP exhibit broader particle size distributions and are shifted toward larger particle size ranges, indicating their different grinding characteristics and particle-packing behavior in the fresh mixtures. Figure 3 presents the XRD patterns of OSP and CBP. The main crystalline phase of OSP is calcite, while CBP is mainly composed of aragonite, indicating that the two marine biogenic calcareous powders have different crystal forms of calcium carbonate. Figure 4 shows the SEM images of OSP and CBP. Both OSP and CBP exhibited layered microstructures, but their morphological characteristics were different. OSP consisted of irregular particles with rough and relatively compact layered surfaces, whereas CBP presented a more porous and hierarchical layered structure with open channels. These differences in particle size, crystal form, and micro-morphology may lead to different effects on water absorption, particle interlocking, rheological behavior, and early structural build-up of 3D-printed WCSA cement composites. These results help explain the role of OSP and CBP in the preparation and printing process. The broader particle size distribution and rough morphology may affect particle packing, water demand, and interparticle friction in the fresh mixtures. In particular, the layered OSP particles mainly contribute to the filling and interlocking effects, while the porous CBP structure may promote water adsorption and structural rebuilding after extrusion. Therefore, the particle size, mineral phase, and morphology of OSP and CBP are closely related to the rheological regulation and shape stability of 3D-printed WCSA cement composites.

### 2.2. Preparation Procedure

Mix proportions are listed in Table 1. In this study, the preparation procedure is as follows: the solid components were first dry-mixed for 2 min to obtain a homogeneous powder blend. HPMC and WRA were dissolved in the mixing water and stirred until a uniform solution was formed. Subsequently, the prepared solution was added to the dry mixture, followed by low-speed mixing for 2 min and high-speed mixing for 2 min. After mixing, the fresh mixture was immediately transferred into the printing barrel to fabricate the printed components.

### 2.3. 3D Printing System

The printing process was carried out using an extrusion-based 3D printer (JianYanHuaCe Technology Co., Ltd., Hangzhou, China). The printer mainly consisted of a material extrusion unit, a printing platform, and a sliding guide rail. After mixing, the fresh WCSA cement composites were immediately loaded into the printing barrel and deposited through the nozzle along the preset printing path. A circular nozzle with an inner diameter of 15 mm was used for extrusion, and the layer thickness was controlled at 20 mm. The printed structure consisted of two layers, with each layer containing five parallel filaments. The printing speed was fixed at 10 mm/s for all mixtures.

### 2.4. Test Methods

All quantitative tests were repeated in three times.

#### 2.4.1. Particle Size Distribution

The particle size distributions of WCSA cement, OSP, and CBP were measured using a laser particle size analyzer (BECKMAN, COULTER’s LS13320, Indianapolis, IN, USA). Before testing, the powder samples were dried and uniformly dispersed to reduce the influence of particle agglomeration on the test results.

#### 2.4.2. Setting Time

The setting time of the WCSA cement composites was measured using a Vicat apparatus. After mixing, the fresh mixture was immediately placed into the Vicat mold, and the surface was gently leveled. The initial and final setting time were recorded to evaluate the influence of OSP and CBP on the early stiffening behavior of the WCSA cement composites. Each mixture was tested three times, and the average value was used for analysis.

#### 2.4.3. Rheological Properties

The rheological properties of 3D-printed WCSA cement composites were measured using a rotary rheometer (HAAKE MARS 40, Thermo Fisher Scientific, Bremen, Germany). The rheological measurements included static yield stress, dynamic yield stress and thixotropic behavior, which were used to evaluate the structural build-up, flow resistance and structural rebuilding ability of the fresh mixtures.

For the static yield stress test, the fresh paste was first loaded into the rheometer and sheared at a high constant shear rate for 2 min to obtain a uniformly mixed state. The shear was then stopped, and the paste was kept at rest for 20 min to allow sufficient structural rebuilding. Subsequently, a low constant shear rate of 0.1 s^−1^ was applied for 2 min, and the shear stress evolution with time was recorded.

The dynamic yield stress consisted of four consecutive stages. In the first stage, the fresh mixture was sheared at a constant shear rate of 100 s^−1^ for 120 s to eliminate the influence of previous shear history and obtain a comparable initial state. Subsequently, the mixture was kept at rest for 120 s to allow partial structural rebuilding. In the third stage, the shear rate was increased from 0 to 200 s^−1^ within 120 s to describe the breakdown behavior of the internal flocculated structure under increasing shear. Finally, the shear rate was decreased from 200 to 0 s^−1^ within 120 s to obtain the flow curve of the mixture after shear-induced structural breakdown. The yield stress and plastic viscosity were obtained by fitting the flow curve using the Bingham model.

A three-stage shear protocol was adopted to evaluate the thixotropic behavior of WCSA cement composites. The paste was first pre-sheared at 100 s^−1^ for 60 s and then kept at rest for 4 min. Subsequently, the apparent viscosity was recorded at a low shear rate of 0.1 s^−1^ for 60 s, followed by shearing at 100 s^−1^ for 30 s to destroy the internal flocculated structure. Finally, the shear rate was reduced to 0.1 s^−1^ and maintained for 60 s. The thixotropic recovery degree of the paste can be calculated as follows:

$$\eta =\frac{\eta_{1}}{\eta_{0}}\times 100\%$$

where η is the thixotropic recovery degree, and η_0_ and η_1_ are the initial apparent viscosity and recovered apparent viscosity, respectively.

#### 2.4.4. Structural Deformation

Structural deformation was used to evaluate the shape stability of the printed structures. After printing, the actual height of the structure was measured, and the deformation rate was calculated as follows: $D\;=\;\frac{\left|L\;-{\;L}_{0}\right|}{{3L}_{0}}\;+\;\frac{\left|W\;-{\;W}_{0}\right|}{{3W}_{0}}\;+\;\frac{\left|H\;-\;H_{0}\right|}{{3H}_{0}}\;\times \;100\%$, where D is structural deformation; W_0_, L_0_, and H_0_ represent the size of the sample; and W, L and H represent the maximum size of the printed specimen.

#### 2.4.5. Mechanical Properties

The mechanical strength of the WCSA cement composites was evaluated by compressive and flexural strength tests using a universal testing machine (MTS, Eden Prairie, MN, USA). The printed specimens were cured under standard curing conditions for 28 d before testing. After curing, the printed components were cut into 40 mm × 40 mm × 160 mm prisms and 40 mm × 40 mm × 40 mm cubes for flexural and compressive strength tests, respectively. For each group, three specimens were tested, and the average value was reported. The mechanical strength tests were conducted at a loading rate of 0.3 kN/s with a measuring range of 10–300 kN.

#### 2.4.6. XRD and SEM

X-ray diffraction (XRD, D8-Advance, Bruker, Karlsruhe, Germany) was used to identify the crystalline phases of OSP and CBP, and the hydration products of the hardened WCSA cement composites. The XRD test was performed using Cu Kα radiation with a wavelength of 0.15418 nm over a 2θ range of 5–80°. Before testing, the hardened samples were crushed, ground into powder, and passed through a 200-mesh sieve. Scanning electron microscopy (SEM, Hitachi Regulus, 8100, Tokyo, Japan) was used to observe the morphology of OSP and CBP, and the microstructure of the hardened samples. The samples were immersed in anhydrous ethanol for 24 h to stop hydration, dried at 40 °C, freeze-dried, and then gold-coated before SEM observation.

## 3. Results and Discussion

### 3.1. Setting Time

**Figure 5:**
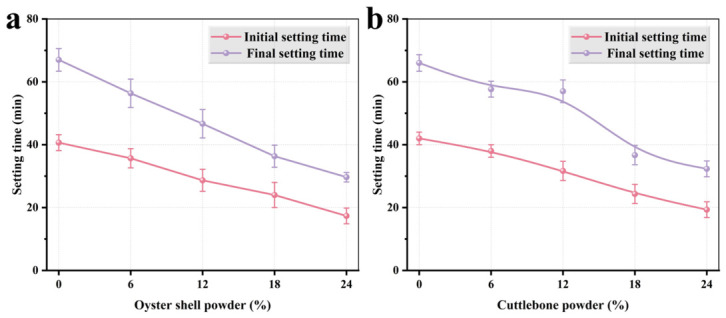
Setting time of 3D-printed WCSA cement composites with different contents of: (**a**) OSP and (**b**) CBP.

Setting time is an essential parameter for evaluating the open time and early structural build-up of WCSA cement paste [27]. For 3D-printed cementitious materials, appropriate setting behavior is required to maintain extrudability during the printing process and provide sufficient shape stability after deposition [28]. As shown in Figure 5, the incorporation of OSP and CBP both shortened the initial and final setting times of WCSA cement paste, and the reduction became more pronounced with increasing OSP and CBP content. As the OSP and CBP contents increased from 0% to 24%, the initial setting time decreased from 41 min to 17 min and from 42 min to 20 min, while the final setting time decreased from 67 min to 30 min and from 66 min to 33 min, respectively. This may be attributed to the filling and nucleation effects of these two marine solid waste powders, which increased the solid phase content and promoted the formation of early hydration products. Meanwhile, the reduction in free water and the enhanced particle contact accelerated the development of the flocculated structure in WCSA cement paste. Therefore, the shortened setting time is favorable for improving shape stability after deposition, but excessive acceleration may narrow the printable window and weaken extrusion continuity or interlayer bonding.

### 3.2. Static Yield Stress

**Figure 6:**
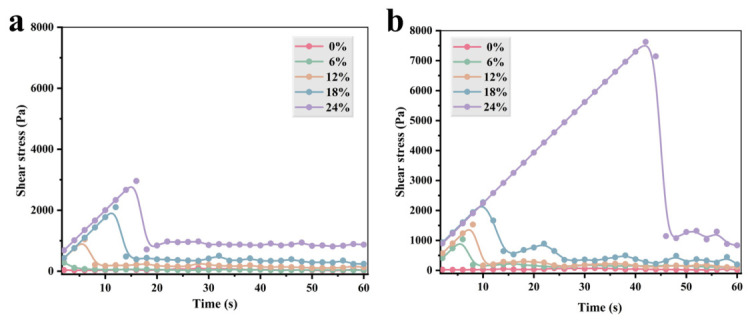
Shear stress of 3D-printed WCSA cement composites with different contents of (**a**) OSP and (**b**) CBP changes with time.

Static yield stress represents the minimum stress required to initiate the flow of paste after resting, and it is commonly used to characterize the early structural build-up of 3D-printed cementitious materials [29]. As shown in Figure 6, the shear stress of WCSA cement composites increased with the increasing OSP and CBP contents. With increasing OSP and CBP contents, the shear stress of WCSA cement composites gradually shifted upward, indicating enhanced resistance to initial flow after resting. This increase became more pronounced at higher powder contents, especially for the 24% CBP mixture, whose shear stress response was much higher than those of the other groups. This phenomenon may be attributed to the increased solid phase content and reduced free water after the addition of marine solid waste powders. Meanwhile, the enhanced particle contacts and filling effect strengthened the internal particle skeleton of the WCSA cement composites matrix.

**Figure 7:**
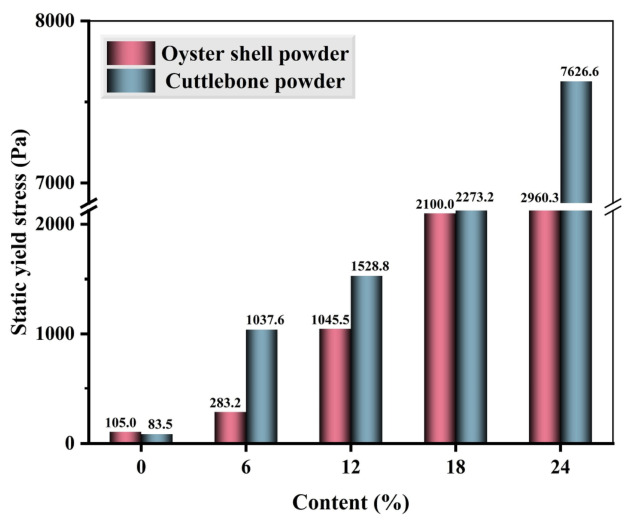
Static yield stress of 3D-printed WCSA cement composites with different contents of OSP and CBP.

Static yield stress is generally obtained from the peak shear stress, which reflects the resistance of fresh paste to initial flow after resting. The calculated static yield stress values are presented in Figure 7. As the OSP and CBP contents increased from 0% to 24%, the static yield stress of 3D-printed WCSA cement composites increased from 105.0 Pa to 2960.3 Pa and from 83.5 Pa to 7626.6 Pa, respectively. At the same dosage, WCSA cement composites with CBP generally produced a higher static yield stress than that with OSP, especially at 24%, indicating that CBP had a stronger effect on accelerating the structural build-up of WCSA cement composites.

### 3.3. Dynamic Yield Stress

**Figure 8:**
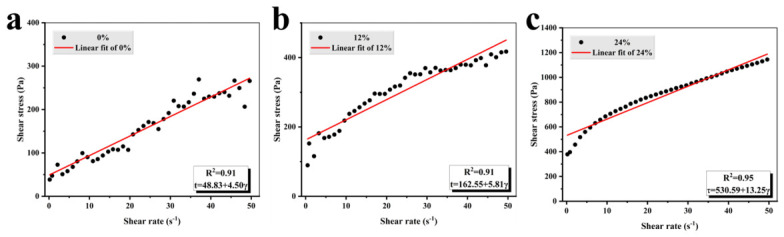
Effect of OSP content on the dynamic yield stress of 3D-printed WCSA cement composites: (**a**) 0%; (**b**) 12%; (**c**) 24%.

**Figure 9:**
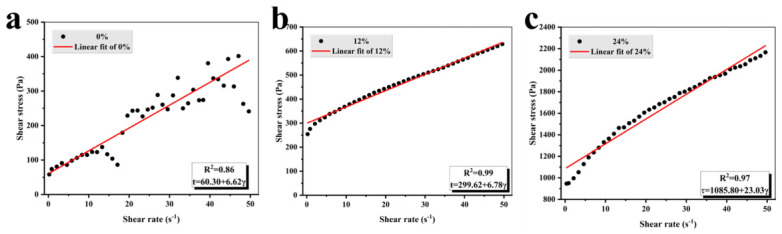
Effect of CBP content on the dynamic yield stress of 3D-printed WCSA cement composites: (**a**) 0%; (**b**) 12%; (**c**) 24%.

The shear stress curves of WCSA cement composites were fitted by the Bingham model to obtain the dynamic yield stress and plastic viscosity. The dynamic yield stress reflects the initial stress required for the paste to maintain flow under continuous shearing, while the plastic viscosity represents the flow resistance after yielding, both of which are closely related to the extrudability of 3D-printed cementitious materials [30]. Figure 8 and Figure 9 show the relationships between shear stress and shear rate in the 3D-printed WCSA cement composites incorporating OSP and CBP. It can be observed that the shear stress of all mixtures increased with increasing shear rate. After the incorporation of OSP and CBP, both the intercept and slope of the fitted curves increased, indicating that the dynamic yield stress and plastic viscosity of the paste were improved. As the OSP and CBP contents increased from 0% to 24%, the dynamic yield stress of 3D-printed WCSA cement composites increased from 48.83 Pa to 530.59 Pa and from 60.30 Pa to 1085.80 Pa, respectively. Meanwhile, the plastic viscosity increased from 4.50 Pa·s to 13.25 Pa·s and from 6.62 Pa·s to 23.03 Pa·s, respectively. Compared with OSP, CBP showed a more significant effect on increasing the dynamic yield stress and plastic viscosity, indicating that CBP had a stronger influence on enhancing the flow resistance of 3D-printed WCSA cement composites. This may be attributed to the porous and layered structure of CBP, which further strengthened water absorption and particle interlocking, resulting in higher shear flow resistance of the paste. The R^2^ of all mixtures were higher than 0.85, indicating that the Bingham model could reasonably describe the relationship of shear stress and shear rate. Therefore, the increase in dynamic yield stress was beneficial for maintaining the shape of the extruded filaments after deposition, while excessive OSP and CBP content may lead to excessive extrusion resistance and thus affect printing continuity.

### 3.4. Thixotropy

**Figure 10:**
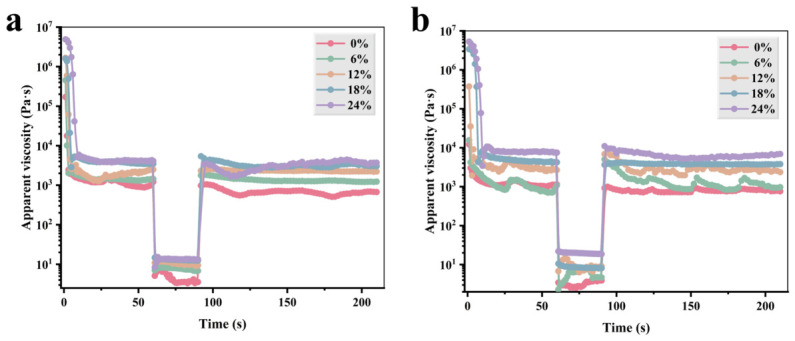
Apparent viscosity variation with time based on the three-stage curves with different contents of: (**a**) OSP and (**b**) CBP.

Thixotropy is an important rheological property used to describe the structural recovery ability of fresh cementitious materials after shearing, and it reflects the transition of paste from a flowable state to a stable structural state [31]. In this study, the thixotropic behavior was determined by a three-stage shear test, in which the apparent viscosity recovery after high-shear breakdown was used to evaluate the structural rebuilding ability of the fresh mixtures. For 3D-printed WCSA cement composites, appropriate thixotropic behavior is beneficial for balancing extrusion flowability and shape stability after deposition. As shown in Figure 10, the three-stage shear curve was used to investigate the apparent viscosity recovery of WCSA paste under dynamic shearing and static recovery conditions. With the increase in OSP and CBP contents, the apparent viscosity of WCSA cement composites generally increased, and the curves gradually shifted upward. With increasing OSP and CBP contents, the apparent viscosity curves gradually shifted upward, and the recovery-stage viscosity increased more clearly at high powder contents. Among all mixtures, the 24% CBP mixture exhibited the highest apparent viscosity, indicating a stronger structural rebuilding ability after shearing.

**Figure 11:**
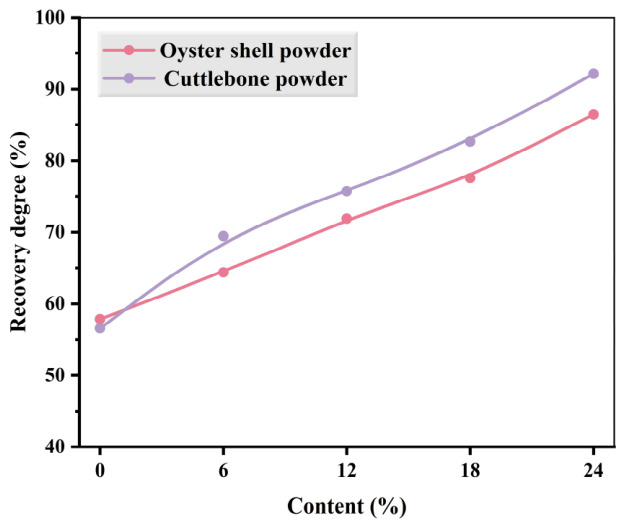
Thixotropic recovery degree of 3D-printed WCSA cement composites.

The thixotropic recovery degree can further characterize the structural rebuilding ability of paste after shearing, and it is an important parameter for evaluating the early structural build-up of 3D-printed cementitious materials. As shown in Figure 11, the thixotropic recovery degree of 3D-printed WCSA cement composites gradually increased with the increase in OSP and CBP contents. As the OSP and CBP contents increased from 0% to 24%, the thixotropic recovery degree of 3D-printed WCSA cement composites increased from 57.89% to 86.46% and from 56.60% to 92.14%, respectively.

### 3.5. Structural Deformation

**Figure 12:**
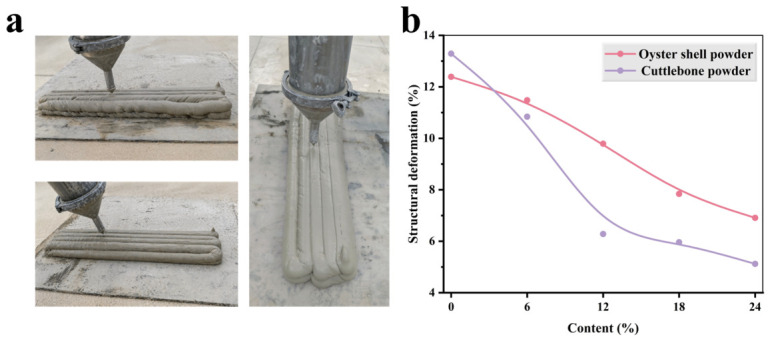
Structural deformation and macroscopic printing morphology of 3D-printed WCSA cement composites: (**a**) structural deformation with different OSP and CBP contents; (**b**) macroscopic printed morphology.

**Figure 13:**
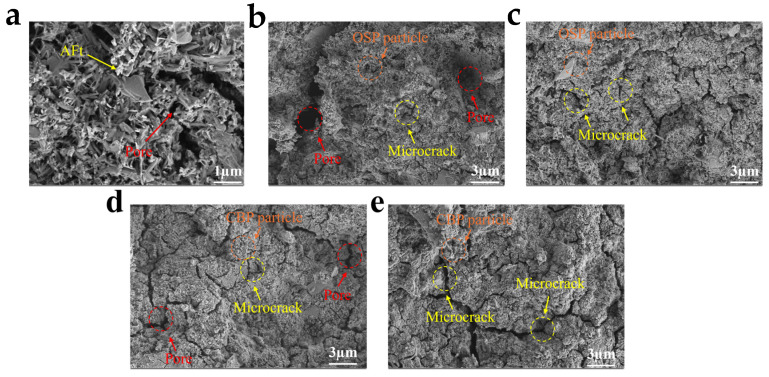
SEM images of 3D-printed WCSA cement composites with different marine calcareous powders: (**a**) 0%; (**b**) OSP-12%; (**c**) OSP-24%; (**d**) CBP-12%; (**e**) CBP-24%.

Structural deformation is an important indicator for evaluating the buildability and shape stability of 3D-printed cementitious materials, as it reflects the ability of printed layers to resist deformation under self-weight after deposition [32]. The structural deformation was calculated according to the method described in Section 2.4.4. Figure 12a presents the macroscopic morphology of 3D-printed WCSA cement composites. As shown in Figure 12b, the structural deformation of WCSA cement composites gradually decreased with the increase in OSP and CBP contents, indicating that both marine calcareous powders could improve the stability of the printed structure. As the OSP and CBP contents increased from 0% to 24%, the structural deformation of 3D-printed WCSA cement composites decreased from 12.39% to 6.91% and from 13.29% to 5.12%, respectively. At the same dosage, the composites containing CBP generally exhibited lower structural deformation than those containing OSP. The decrease in structural deformation rate was closely related to the improved rheological properties of WCSA paste. The increased yield stress enhanced the resistance of deposited filaments to self-weight deformation, while the improved thixotropic recovery promoted rapid structural rebuilding after deposition. Therefore, OSP and CBP effectively reduced the structural deformation rate of printed filaments. SEM observations further supported the structural deformation results. As shown in Figure 13, 24% powder contents promoted hydration product accumulation and particle packing, which was consistent with the reduced structural deformation and improved shape stability of printed filaments.

### 3.6. Mechanical Strength

**Figure 14:**
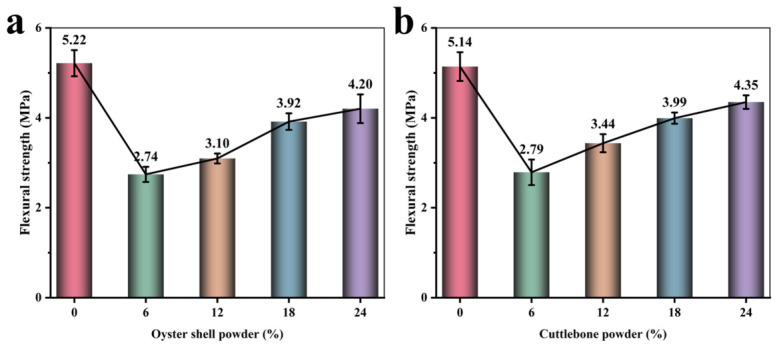
Flexural strength of 3D-printed WCSA cement composites with different contents of: (**a**) OSP and (**b**) CBP.

Flexural strength is an important parameter for evaluating the resistance of 3D-printed WCSA cement composites to bending failure, and it is closely related to interlayer bonding and hardened structural stability. As shown in Figure 14, after the incorporation of OSP and CBP, the flexural strength of the mixtures was generally lower than that of the control group, but it gradually recovered within the range of 6–24% contents. The flexural strength of 3D-printed WCSA cement composites with OSP increased from 2.74 MPa at 6% content to 4.20 MPa at 24% content, while that with CBP increased from 2.79 MPa to 4.35 MPa. Compared with OSP, the flexural strength of 3D-printed WCSA cement composites with CBP exhibited slightly higher flexural strength in the contents of 12–24%, indicating that CBP had a more pronounced effect on recovering the flexural performance of the hardened matrix.

**Figure 15:**
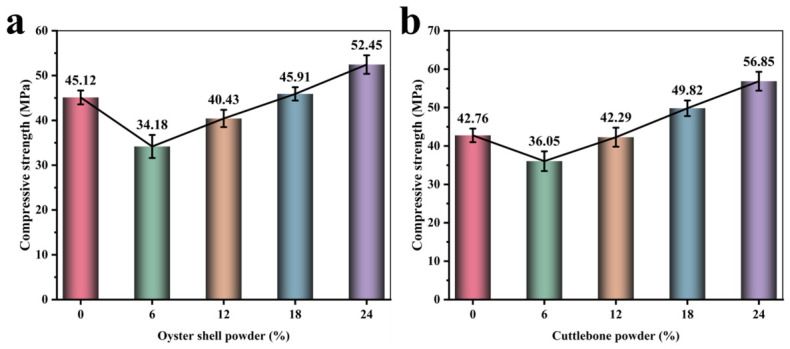
Compressive strength of 3D-printed WCSA cement composites with different contents of: (**a**) OSP and (**b**) CBP.

Compressive strength mainly reflects the compactness and overall load-bearing capacity of the hardened matrix, and it is an important indicator for evaluating the mechanical performance of 3D-printed cementitious composites. As shown in Figure 15, the variation trend of compressive strength for the OSP and CBP was generally consistent with that of flexural strength. After powder incorporation, the compressive strength decreased compared with the control group, but gradually increased as the OSP and CBP contents increased from 6% to 24%, rising from 23.68 MPa to 30.76 MPa and from 24.79 MPa to 31.63 MPa, respectively. This phenomenon may be attributed to the limited cementitious activity of OSP and CBP, which weakened the continuity of the WCSA hydration matrix and interlayer bonding. With increasing powder content, the higher yield stress and thixotropic recovery enhanced the structural build-up of fresh paste, while the reduced structural deformation and filling effect improved the stability and compactness of the printed matrix, leading to the gradual recovery of flexural and compressive strengths.

### 3.7. Mechanism Analysis

**Figure 16:**
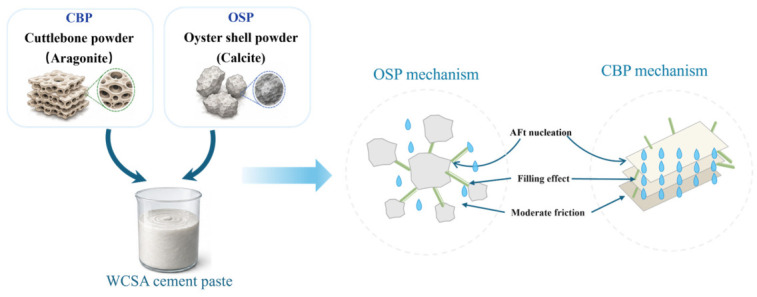
Proposed rheological regulation mechanism of OSP- and CBP-modified 3D-printed WCSA cement composites.

As shown in Figure 16, OSP and CBP improved the rheological properties and shape stability of 3D-printed WCSA cement composites mainly through the filling effect, water absorption, particle interlocking, and early structural build-up. In this study, the increased yield stress, plastic viscosity, and thixotropic recovery degree, together with the reduced structural deformation, were consistent with the reported structural build-up behavior of 3D-printed cementitious materials. Xu et al. [13] also indicated that admixtures could improve the printability of white cementitious materials by regulating rheological parameters and early structural formation.

The different effects of OSP and CBP were mainly related to their particle size, crystal form, and micro-morphology. The irregular and layered calcite particles of OSP enhanced the filling effect and particle interlocking, which is consistent with Seifu et al., who reported that OSP affected carbonate-containing hydration products in CSA-containing cementitious systems [20]. In contrast, the porous and layered aragonite structure of CBP resulted in a stronger effect on rheological regulation and shape stability, which agrees with Yang et al. regarding the hierarchical porous calcium carbonate structure of cuttlebone [18,19,21]. Although OSP and CBP reduced the mechanical strength due to their limited cementitious activity, the strength gradually recovered at higher powder contents because of the enhanced filling effect, improved particle packing, and reduced structural deformation.

## 4. Conclusions

In this study, oyster shell powder (OSP) and cuttlebone powder (CBP) were incorporated into 3D-printed white calcium sulphoaluminate (WCSA) cement composites, and their effects on rheological behavior, structural build-up stability, shape retention and mechanical properties were investigated. The incorporation of OSP and CBP improved the buildability and dimensional stability of 3D-printed WCSA cement composites, while also demonstrating the potential of marine calcareous powders for sustainable cementitious materials. The main conclusions are summarized as follows:

(1) OSP and CBP shortened the setting time of 3D-printed WCSA cement composites and improved printing operability. As the contents increased from 0% to 24%, the initial setting time decreased from 41 min to 17 min and from 42 min to 20 min, while the final setting time decreased from 67 min to 30 min and from 66 min to 33 min, respectively.

(2) OSP and CBP significantly improved the rheological properties of 3D-printed WCSA cement composites. As the content increased to 24%, the static yield stress increased to 2960.3 Pa and 7626.6 Pa, respectively, while the dynamic yield stress and plastic viscosity also increased, indicating enhanced flow resistance and structural build-up ability. This effect was more pronounced for CBP due to its porous and layered structure.

(3) The addition of OSP and CBP improved the thixotropic recovery and reduced the structural deformation of 3D-printed WCSA cement composites. The increase in thixotropic recovery degree confirmed the enhanced structural rebuilding ability after shearing, while the reduction in structural deformation from 12.39% to 6.91% and from 13.29% to 5.12% verified the improved shape stability of printed filaments during stacking.

(4) The addition of OSP and CBP reduced the mechanical strength compared with the control group, but the strength gradually recovered within 6–24%. SEM observations supported this result, showing that higher powder contents improved particle packing and matrix compactness, while local pores and microcracks still limited strength development.

## Figures and Tables

**Table 1:** Mix proportion of 3D-printed MPCCs (% by mass).

WCSA	Water	HPMC	WRA	TA	Bentonite	Quartz Sand	OSP	CBP
100	35	0.1	0.2	0.2	1	40	0, 6, 12, 18, 24	-
100	35	0.1	0.2	0.2	1	40	-	0, 6, 12, 18, 24

## Data Availability

The original contributions presented in this study are included in the article. Further inquiries can be directed to the corresponding authors.
